# miR-146a is a critical target associated with multiple biological pathways of skin aging

**DOI:** 10.3389/fphys.2024.1291344

**Published:** 2024-02-29

**Authors:** Klodjan Stafa, Antonella Rella, Whitby Eagle, Kelly Dong, Kelsey Morris, Dawn Layman, Krystle Corallo, Jacqueline Trivero, Robert Maidhof, Earl Goyarts, Nadine Pernodet

**Affiliations:** ^1^ Research and Development, The Estée Lauder Companies, Melville, NY, United States; ^2^ Estée Lauder Research Laboratories, Melville, NY, United States

**Keywords:** aging, circadian rhythm, epigenetic, miR-146a-5p, skin

## Abstract

**Introduction:** The skin is the largest organ of the human body and fulfills protective, immune, and metabolic functions. Skin function and barrier integrity are actively regulated through circadian rhythm–associated genes and epigenetic mechanisms including DNA methylation/demethylation, histone acetylation/deacetylation, and microRNAs. MicroRNA-146a-5p (miR-146a) has been associated with immune activation and skin inflammation; however, the role of miR-146a in regulating skin aging is an open question. This study investigated the role of miR-146a in fibroblasts obtained from different donors in the context of aging, and a potential association of this miRNA with circadian rhythm.

**Methods:** Normal human dermal fibroblasts (NHDFs) from 19y, 27y, 40y, and 62y old donors were used to analyze for miR-146a expression. Expression of miR-146a was downregulated with the hsa-mirVana miR-146a inhibitor, and upregulated with an extract from Adansonia digitata. Effects on markers of skin aging, including cell proliferation, production of Collagen-1 and inflammatory cytokines were assessed.

**Results:** We show that the expression of miR-146a decreases with age in dermal fibroblasts and inhibition of miR-146a in 19y and 62y old NHDFs induced significant changes in essential clock genes indicating an association with circadian rhythm control. Furthermore, downregulation of miR-146a results in a reduction of cellular proliferation, Collagen-1 production, as well as an increase in DNA damage and pro-inflammatory markers. Activation of miR-146a with the Adansonia digitata extract reduced the deleterious effects seen during miR-146a inhibition and increased miR-146a transport through exosome transfer.

**Conclusion:** miR-146a interacts with multiple biological pathways related to skin aging, including circadian rhythm machinery, cell-to-cell communication, cell damage repair, cell proliferation, and collagen production and represents a promising target to fight skin aging. Adansonia digitata extract can promote miR-146a expression and therefore support skin cells’ health.

## Introduction

The skin is the first barrier against exposure to environmental insults, including ultraviolet irradiation and particulate pollution like cigarette smoke (Valacchi et al., 2012). As other organs, the skin is tightly regulated by 24 h oscillations of daytime and nighttime cycles called circadian rhythm, a network well conserved throughout evolution, entrained by the master clock located in the suprachiasmatic nucleus (SCN) within the hypothalamic region of the brain. This metabolic machinery controls peripheral clocks located in mostly every cell of the body with strictly timed cellular functions supporting skin protection during the day and skin repair at night (Matsui et al., 2016). At the cellular-molecular level, the circadian clocks are finely governed by interdependent feedback loops involving transcription and translation shuffling between daytime clock genes, Clock/Bmal-1, and nighttime clock genes, Period (Per) 1-3/Cryptochrome (Cry) 1-2 (Matsui et al., 2016). Circadian rhythms can be altered by external and internal factors, including physiologic aging (Hood and Amir, 2017), jetlag, presence of light at night (Dong et al., 2019) and lack of sleep.

Environmental and lifestyle factors are linked to genetic activity and cellular functions through epigenetics, a process by which the expression of genes is regulated through modifications to chromatin or to messenger RNA processing, but the DNA sequence is not altered (Pal and Tyler, 2016). Whereas only 20%-30% of aging is attributable to the genetic code itself, the majority of the remaining processes responsible for aging are determined by epigenetic changes (Pal and Tyler, 2016). Importantly, epigenetic changes are often reversible, providing the opportunity to develop technologies and treatments that may ameliorate or delay the manifestations of these changes (Pal and Tyler, 2016).

Epigenetic can involve DNA methylation, histone modifications, and microRNA (miRNA) regulation (Pal and Tyler, 2016). miRNAs are a large class of post-transcriptional regulators of gene expression of ∼22 nucleotides long, found in all living organisms, including humans. They predominantly bind the 3′-untranslated region (UTR) of messenger RNA (mRNA) and inhibit translation of mRNA into functional proteins either by cleavage of the mRNA or by reducing the functioning of ribosomes (O’Brien et al., 2018; O’Connell et al., 2012; Lee et al., 1993).

miRNAs are potent and highly tissue-specific regulators of differentiation, maintenance of tissue identity, tissue remodeling, and inflammation. Published evidence also suggests the ability of some miRNAs to affect neighboring cells via exosome transport (Olsen and Ambros, 1999). Expression patterns of miRNAs vary, with several, including miR-155 and miR-146a-5p (miR-146a), being upregulated by NF-κB, and linked to immune activation and skin inflammation (O’Brien et al., 2018; Salido-Guadarrama et al., 2014). miR-146a in particular is upregulated in inflammatory dermatoses, including atopic dermatitis and psoriasis, in which it reduces expression of pro-inflammatory factors, limits recruitment of inflammatory cells, and aids in the resolution of inflammation (Boldin et al., 2011; Rebane et al., 2014).

Besides inflammation, miR-146a has also been linked to other critical pathways relevant to skin aging such as sleep quality, DNA repair/damage, cellular proliferation, and circadian rhythm (Wang et al., 2014; Srivastava et al., 2017). Indeed, chronic inflammation and dysregulation of circadian rhythmicity have been reported in diabetic retinopathy, with decreased levels of miR-146a and loss of Per-1 rhythmicity in diabetic patients (Srivastava et al., 2017). Additionally, a study conducted in healthy participants that were divided in two groups, normal nightly sleep (≥7 h night) and short nightly sleep (<7 h night) monitored different circulating miRNAs, found that miR-146a was significantly decreased in the short nightly group suggesting that habitual insufficient sleep may be associated with circadian rhythm disruption and an increase inflammatory burden that could lead to cardiovascular disorders and co-morbidities (Wang et al., 2014). Modifications to epigenetic regulation can negatively or positively influence the aging process (Hijmans et al., 2019) in the skin; for example, impacting loss of skin progenitor cell identity and self-renewal capability, keratinocyte and fibroblast senescence, increased DNA damage, and heightened immune activation (Hijmans et al., 2019). However, the role of miRNAs in the skin and their effects on skin aging are not well understood. As a motivation for this study, we previously evaluated the expression of miRNAs in skin cells to determine which ones were associated with aging as well as circadian rhythm, thereby identifying miR-146a as a lead candidate. We subsequently investigated whether miR-146a is associated with biological phenomena relevant to the aging process including DNA damage, proliferation, Collagen-1 synthesis, and inflammation.

To ameliorate the detrimental effect of miR-146a downregulation, we treated skin fibroblasts with an extract derived from the Baobab tree (Adansonia digitata). Baobab has a variety of biological properties including antiviral, antioxidant, and anti-inflammatory that would potentially counteract the skin aging process and help with renewal.

## Methods

### Cell culture

Normal human dermal fibroblasts (NHDFs) originating from 19y, 27y (Coriell Institute, Camden, NJ), 40y and 62y old donors (Zen Bio, Durham, NC) were plated and incubated at standard conditions (37°C/5% CO₂). NHDFs were grown in Dulbecco Modified Eagle Medium (DMEM) media (Life Technologies) supplemented with 10% bovine Calf Serum (ThermoFisher) and 1% Penicillin/Streptomycin (Corning, Cat#30-001-Cl) at approximately passage 9 (Olsen and Ambros, 1999).

### Live cells imaging of miR-146 in NHDFs

NHDFs from 19y, 27y, 40y and 62y old donors were plated onto 6-well dishes at a density of 10,000 cells/well, incubated at standard conditions overnight and grown in DMEM media supplemented with 10% Bovine Calf Serum and 1% Penicillin/Streptomycin. The next day, cells were incubated with SmartFlare™ RNA Detection Probe miR-146a (Merck Millipore, SmartFlare™ probe miR-146a, Cat# SF-500). Live cells were imaged on the Nikon A1 Confocal microscope using a 20X objective.

### Quantitative Real-Time PCR (qRT-PCR)

NHDFs from 19y and 62y old donors were plated onto 60 mm dishes at a density of 500,000 cells/dish for 24 h. The next day, the cells were treated with hsa-miR-146a mirVana Inhibitor (Ambion, Cat#4464084) and Dharmafect#3 Transfection Reagent (GE Life Sciences, Cat#T-2003-02) or only with Dharmafect#3 Transfection Reagent. 24 h post-treatments the isolation and the quantification of the RNA was performed with miRNeasy Micro Kit (Qiagen, Cat#217084), and Quant-it™ RiboGreen RNA Assay Kit (Invitrogen, Cat#R11490), respectively. 1ug of RNA was reverse transcribed into complementary DNA (cDNA) using a SuperScript™ IV VILO™ Master Mix (ThemoFisher, Cat#11756050) or TaqMan microRNA Reverse Transcription Kit (ThemoFisher, Cat#4366596). The resulting cDNA was amplified using a Quant Studio 7 Flex system (Applied Biosystems) with a universal PCR master mix (Life Technologies, Cat# 4440040) and the recommended PCR conditions for quantitative assessment of gene transcript levels in the samples.

miR-146a-5p (miR-146a) expression was evaluated in 19y and 62y old NHDFs treated or not treated with different concentration of Adansonia digitata for 24 h in DMEM and gene expression levels of miR-146a (Life Technologies, Cat# 000468) were normalized to an endogenous control RNU44 (Life Technologies, Cat# 001094). miR-146a expression was also evaluated in 21y, 27y and 40y old Chinese NHDFs using miR-146a and RNU44 as endogenous control.

To assess the expression of clock genes, Bmal-1, Per-1 and Clock, in 19y and 62y old NHDFs, Taqman assay probes Per-1 (Hs01092604_m1), ARNTL (Bmal-1, Hs00154147_m1) and Clock (Hs00231857_m1) were used. To evaluate the expression of Lin-28a Taqman probe Lin-28a (Hs01013729_m1) was used. The data were normalized to Glyceraldehyde-3-Phosphate Dehydrogenase control (GAPDH, Hs99999905_m1). qRT-PCR data were calculated using the 2^−ΔΔCT^ method (Livak and Schmittgen, 2001).

### Cell proliferation assays

The effect of miR-146a inhibition on cellular proliferation was assessed by comparing cell proliferation of 19y and 62y old NHDFs treated with hsa-miR-146a mirVana Inhibitor (100 nM; Ambion) and Dharmafect#3 (GE Life Sciences) in DMEM to 19y and 62y old NHDFs treated with Dharmafect#3 alone in DMEM. The effect of miR-146a inhibition on cellular proliferation was also assessed in keratinocytes.

To assess the effect of Adansonia digitata extract on cellular proliferation in older cells, 62y old NHDF were seeded at a density of 20,000 cells/well in a 24-well plate and incubated overnight. Then, the cells were treated with 2% of Adansonia digitata extract once 1 day after plating. Cells were then counted every 24 h for 7 days with a Vi-CELL XR Cell Viability Analyzer (Beckman Coulter).

### Pro-inflammatory cytokines and pro-collagen type I ELISA

19y and 62y old NHDFs were cultured as described above and supernatants were collected at the designated timepoints, and frozen at −80°C until ready to use. Collected supernatants were assayed as per manufacturer’s instructions, using the Milliplex MAP Human Cytokine/Chemokine Magnetic Bead Panel (EMD Millipore, Cat# HCYTOMAG-60K-PX29), Pro-collagen type I C-peptide (PIP) (Takara Bio, Cat #MK101) and Milliplex^®^ Human MMP Magnetic Bead Panel 2—Immunology Multiplex Assay (Millipore Sigma, Cat# HMMP2MAG-55K) kits.

### DNA damage via comet assay

10,000 cells/well of 19y and 62y old NHDFs were plated onto 60 mm dishes and treated with hsa-miR-146a mirVana Inhibitor and Dharmafect#3 or Dharmafect#3 alone or Adansonia digitata or a media control (Untreated Ctrl). Cells were washed with PBS and incubated with PBS-glucose (Thermo Fisher Scientific) for 25 min in the dark. The cells were irradiated with ultraviolet light (5 J/cm^2^ UVA + 40 mJ/cm^2^ UVB) using the Dr. Gröbel irradiation chamber and treated again, as previously described. After 1 h, 4 h or 6 h post treatment, NHDFs were trypsinized, washed and suspended in PBS at 1 × 105 cells/mL. Cells were then dispersed in melted agarose (Trevigen; Gaithersberg, MD) at 37°C at a 1:10 ratio. 75µL of the cell/agarose mixture was pipetted evenly onto each spot of the comet slide (Trevigen) and then incubated at 4°C for 10 min. Slides were immersed in cold lysis solution (Trevigen) on ice overnight. Slides were removed from the lysis solution and placed into an alkaline solution (300 mM NaOH, 1 mM EDTA, pH > 13) at room temperature (RT) for 30 min. Then, the slides were placed in the CometAssay ESII Electrophoresis System. Cold alkaline electrophoresis solution (200 mM NaOH, 1 mM EDTA, pH > 13) was poured into the apparatus. After 30 min at 23 V, the slides were rinsed in H_2_O and immersed in 70% ethanol for 5 min. Slides were removed from the ethanol solution and placed on a towel to air dry overnight. SYBR gold (ThermoFisher) was diluted in TE buffer (10 mM Tris-HCl, 1 mM EDTA, pH 7.5) 1:30000. 100 μL of diluted SYBR gold was pipetted onto each spot. Slides were incubated at RT for 30 min. Slides were viewed under the Keyence BZ-X710 microscope with the FITC filter with the 20X objective. The tail moments were determined with the Comet Score software from Tri Tek.

### ViewRNA in situ hybridization

19y and 62y old NHDFs were seeded in an 8-well tissue culture treated glass slide (Falcon, Cat# 354118) at 20,000 cells/well. Cells were incubated at standard conditions in cell culture media. After 24 h, cells were treated with 2% vol/vol of Adansonia digitata in DMEM or a media control for 48 h, with treatment refreshed at 24 h. Following treatment, miR-146a was labeled *in situ* using the ViewRNA Cell Plus Assay from ThermoFisher (Cat#88-19000-99), as per manufacturers’ protocol, with the appropriate target probe set for miR-146a (Cat#VM1-10253-06). To summarize, cells were first fixed and permeabilized. Next, cells were incubated in a blocking solution. Cells were then incubated with a solution of the miR-146a targeting probe, followed subsequently by solutions with PreAmplifier probes, Amplifier probes, and finally Type 1 Label probes that are conjugated to the Alexa Fluor 546 synthetic dye. Cell nuclei were then stained using a DAPI solution. Cells were mounted in Prolong Glass mounting medium (ThermoFisher, Cat#P36982) with a No. 1.5 cover slip (Corning, Cat#2980-245), and allowed to cure for at least 24 h at RT prior to image acquisition. All images were captured on a Nikon A1-HD 25 laser scanning confocal microscope using a 60×, 1.4 NA oil immersion objective.

### Immunofluorescence

19y and 62y old NHDFs were treated with a proprietary complex of actives including Adansonia digitata (Adansonia digitata complex) in DMEM or a media control for 72 h, with treatment refreshed every 24 h. Following the 72 h treatment, cells were rinsed with PBS, and then fixed for 20 min at RT with a solution of 2% formaldehyde (Thermo Scientific, Cat#28908) in PBS. Cells were washed 3 times in PBS, then permeabilized for 10 min at RT with 0.2% Triton X-100 (Fisher BioReagents, Cat#9002-93-1) in PBS. Cells were blocked for 30 min in a solution comprised of 10% Fetal Bovine Serum (HyClone, Cat#SH30071.03), 0.1% Glycine (Sigma, Cat#G7126), and 3% fraction 5 BSA (Roche, Cat#10735078001) in PBS. Cells were stained for Collagen-1 by incubation in a 1:500 solution of rabbit polyclonal Collagen-1 antibody (Abcam, Cat#ab34710) prepared in PBS supplemented with 2% fraction 5 BSA and 0.1% Triton X-100 for 2 h at RT, followed by a 1 h incubation in a 1:1000 solution of Goat anti-Rabbit IgG (H + L) Alexa Fluor Plus 555, highly cross-adsorbed secondary antibody (Thermo Fisher, Cat#A32732). Cell nuclei were stained with 10 min incubation at RT in NucBlue Fixed Cell Stain ReadyProbes reagent in PBS (Invitrogen, Cat#R37606). Finally, cells were mounted in Prolong Glass mounting medium (Thermo Fisher, Cat#P36982) with a No. 1.5 cover slip (Corning, Cat#2980-245) and allowed to cure for 24 h at RT prior to image acquisition. All images were captured on a Nikon A1-HD 25 laser scanning confocal microscope using a 60×, 1.4 NA oil immersion objective. Image analysis was done in the Nikon Elements Advanced Research software.

### Exosomal transport of miR-146a

19y old NHDF were seeded in DMEM media in 100 mm petri dishes at 70% confluency. The next day, 19y old NHDF were treated with 2% Adansonia digitata in exosome depleted FBS media (ThermoFisher, Cat#A3381901) or only with exosome depleted FBS media. Treatment was continued for 48 h, and then conditioned supernatants were transferred to naïve 62y old NHDF. After 24 h, 62y old NHDF cells were harvested, RNA was extracted, reverse-transcribed and qRT-PCR was performed to identify the non-exosomal miR-146a expression level, whereas the supernatants were collected and centrifugated at 2000 × g for 30 min to remove cell debris. Collected supernatants were mixed with exosome isolation reagent (ThermoFisher, Cat#4478359), and kept at 4°C overnight. Exosome extraction was performed as per manufacturer guidelines (ThermoFisher, Cat#4478545). RNA was extracted, reverse-transcribed and qRT-PCR was performed to identify the exosomal miR-146a expression level.

### Statistical analysis

Paired t tests were used to compare cell counts at different ages. The effect of age on cell proliferation was assessed with a 1-way ANOVA with Tukey post hoc test. Statistical significance of differences between means for miR-146a inhibition was performed using 2-way ANOVA followed by Dunnett post-hoc test. Differences between means for Adansonia digitata extract administration were evaluated using an unpaired t-test with a 2-tailed unpaired t-test and 95% confidence interval on day 7. Statistical analyses were performed using Prism 7.0 (GraphPad). Standard error in cellular proliferation assays was calculated in Excel (Microsoft).

## Results

As part of a previously conducted miRNAs screen, we identified miR-146a-5p (miR-146a) as being differentially expressed with age and being implicated in a loss of synchronization with circadian rhythm in fibroblasts.

### miR-146a expression in human fibroblasts decreases with age

**FIGURE 1:**
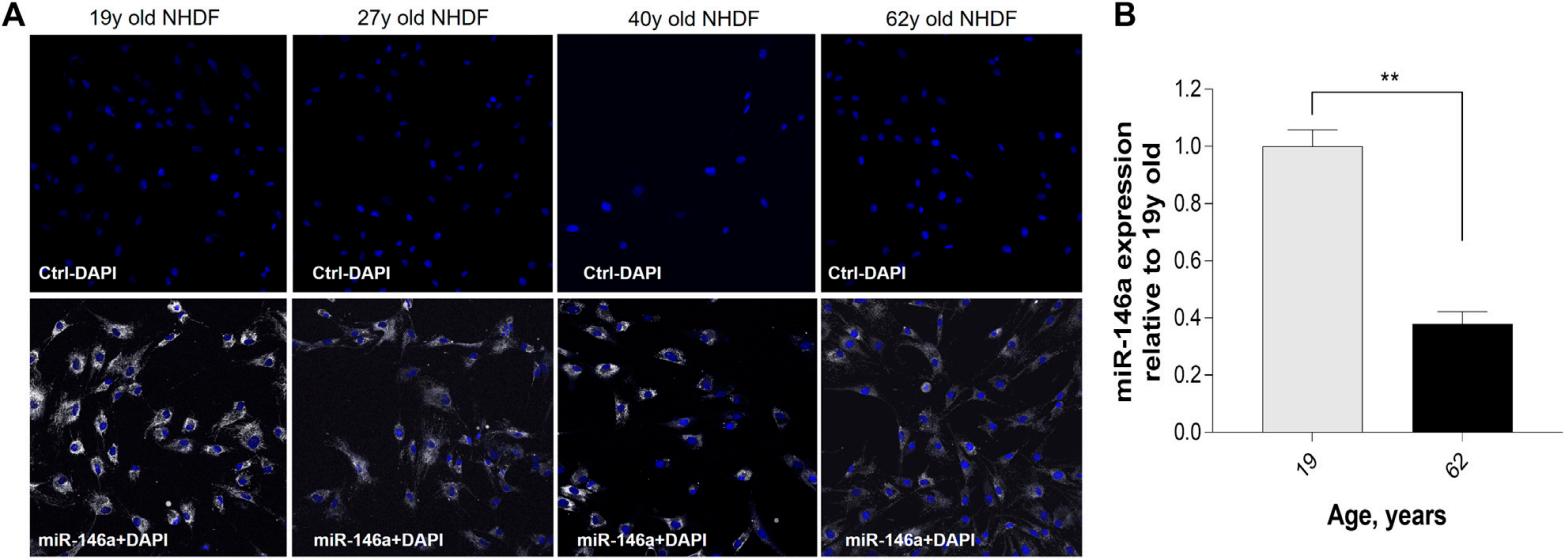
Expression of miR-146a in NHDFs (normal human dermal fibroblasts) decreases with age. **(A)** Detection of miR-146a (white) with SmartFlare™ RNA Detection Probe in 19y, 27y, 40y, and 62y old NHDFs. **(B)** miR-146a expression in 62y old NHDF relative to 19y old NHDF assayed by qRT-PCR. **p = 0.0001; Error bars ± SEM; qRT-PCR, quantitative Real-Time Polymerase Chain Reaction. Data are representative of at least two independent experiments.

To evaluate miR-146a expression in NHDFs, we used the SmartFlare™ RNA Detection Probe miR-146a. The probe is designed to fluoresce when it detects specific targets in live cells. miR-146a staining in 19y, 27y, 40y, and 62y old Caucasian NHDFs demonstrated that it is highly expressed and widely distributed throughout younger cells. Expression of miR-146a is decreased among aging cells compared to the 19y old NHDF (Figure 1A). This decrease was demonstrated by qRT-PCR (Figure 1B). In addition, we evaluated miR-146a expression in 21y, 27y and 40y old Chinese NHDFs by qRT-PCR and a similar pattern to Caucasian NHDFs was observed (Supplementary Figure S1).

### Effects of miR-146a inhibition on circadian machinery and skin aging pathways

#### miR-146a inhibition and circadian machinery

**FIGURE 2:**
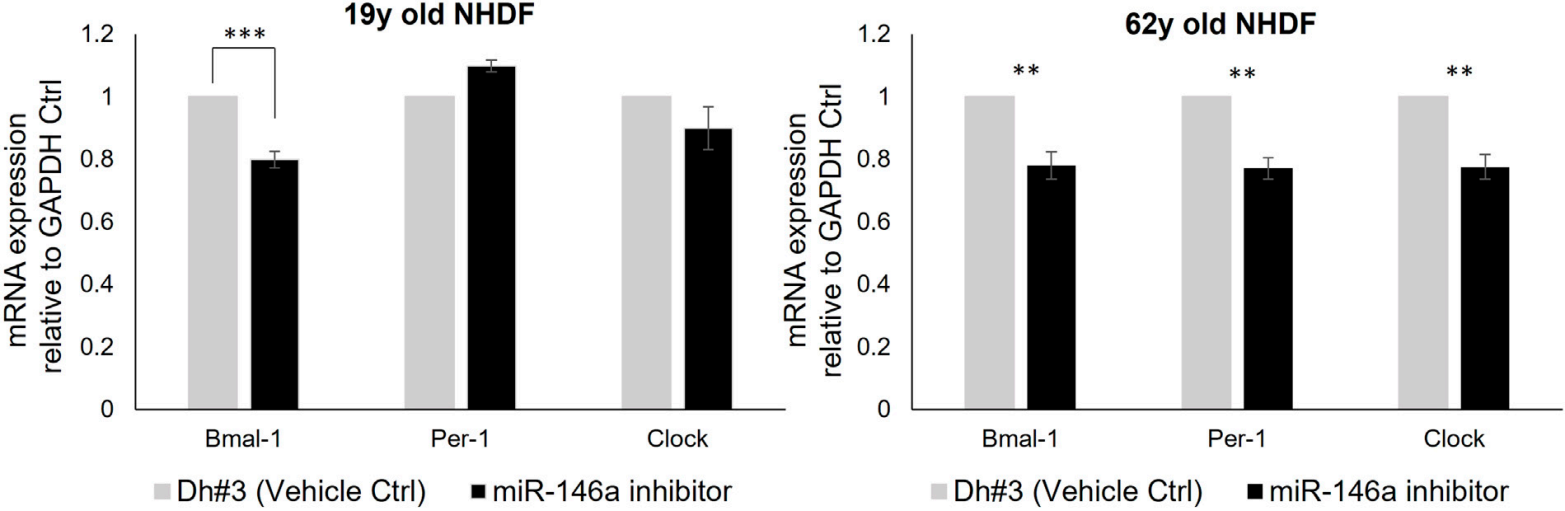
miR-146a inhibition impacts the clock gene machinery in NHDFs. miR-146a inhibition in 19y old NHDF leads to a decrease of Bmal-1 and in 62y old NHDF to a decrease of Bmal-1, Per-1 and Clock assayed by qRT-PCR. **p = 0.001, ***p = 0.0001. Dh#3, Dharmafect #3; Error bars ± SEM; Data are representative of at least two independent experiments.

To explore the interaction between miR-146a and the circadian clock machinery, we inhibited miR-146a expression (Supplementary Figure S2) and evaluated Bmal-1, Per-1 and Clock gene expression by qRT-PCR. A decrease of Bmal-1, Per-1 and Clock expression was observed in 62y old NHDF, whereas only a significant decrease of Bmal-1 expression was observed in 19y old NHDF (Figure 2). This shows the interaction between miR-146a and circadian machinery. We then followed hallmarks of skin cell aging to evaluate the impact of the loss of miR-146a.

#### Cellular proliferation

**FIGURE 3:**
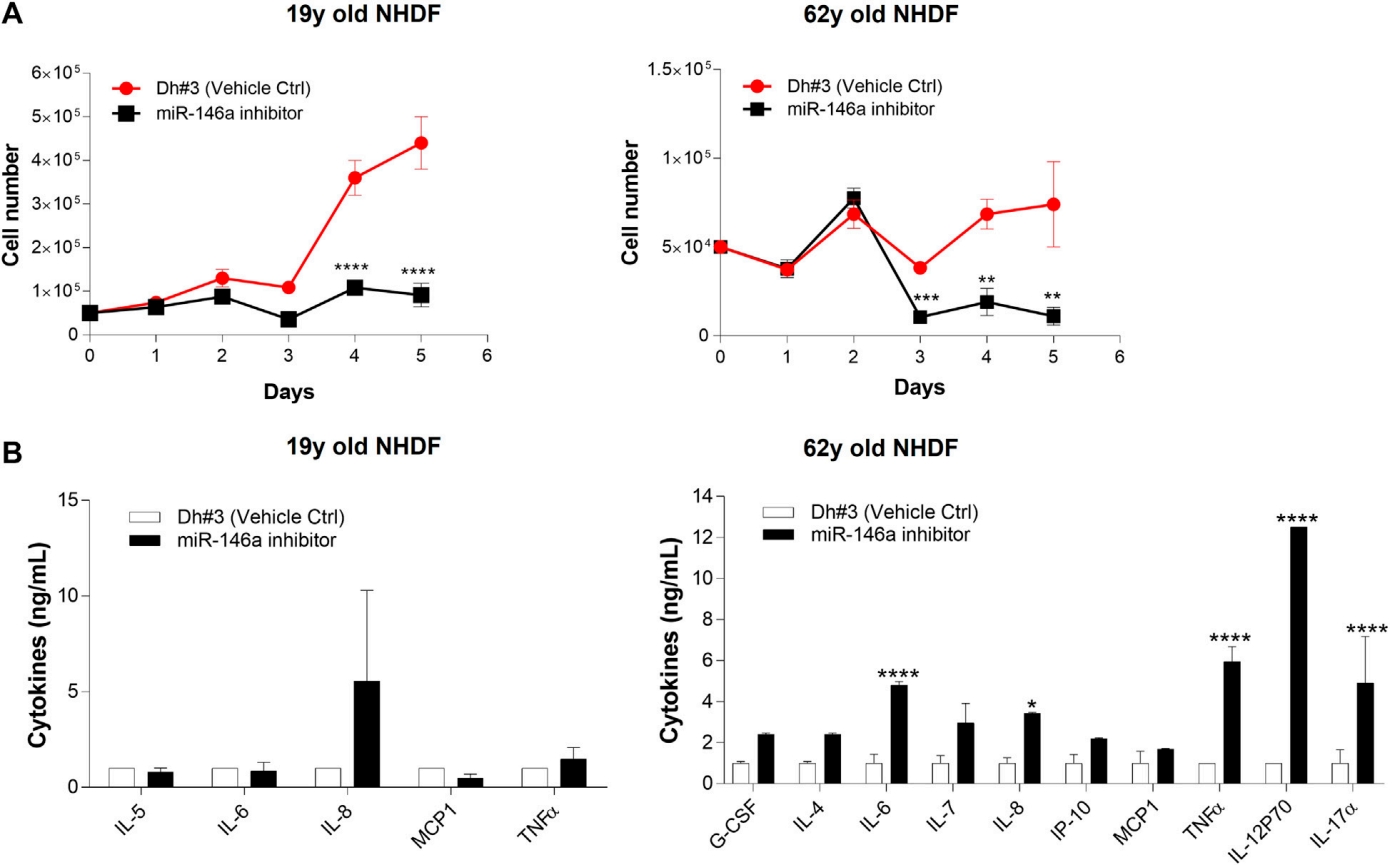
**(A)** miR-146a inhibition impacts cellular proliferation in NHDFs. Cell count of 19y old and 62y old NHDFs subjected to 5 days of miR-146a inhibition. Cell count was performed each day post treatment on both Dh#3 (Vehicle Ctrl) and miR-146a inhibitor-treated samples. **(B)** miR-146a inhibition impacts cytokines production in NHDFs. Cytokines analysis of the spent media from both 19y old and 62y old NHDFs subjected to 5 days of miR-146a inhibition. Error bars ± SEM are plotted for both cell lines. **p = 0.01, ***p < 0.001, ****p < 0.0001. Dh#3, Dharmafect #3; Data are representative of at least two independent experiments.

The effect of miR-146a inhibition on cellular proliferation was measured daily for a period of 5 days using trypan blue staining. Inhibition of miR-146a resulted in growth arrest becoming prominent on day 3 in both 19y and 62y old NHDFs (Figure 3A). Similar results were observed when miR-146a was inhibited in adult keratinocytes (Supplementary Figure S3).

#### Pro-inflammatory cytokines

Next, we evaluated markers related to the inflammatory machinery in 19y and 62y old donors and showed that when miR-146a is inhibited, there is a significant increase of several pro-inflammatory cytokines and chemokines, especially in the 62y old donor (Figure 3B). Our findings that miR-146a inhibition increases a variety of pro-inflammatory mediators corroborate previous evidence that miR-146a plays an important role in inflammation control (O’Connell et al., 2012; Rebane et al., 2014).

#### Pro-collagen type I and metalloproteinases (MMPs)

**FIGURE 4:**
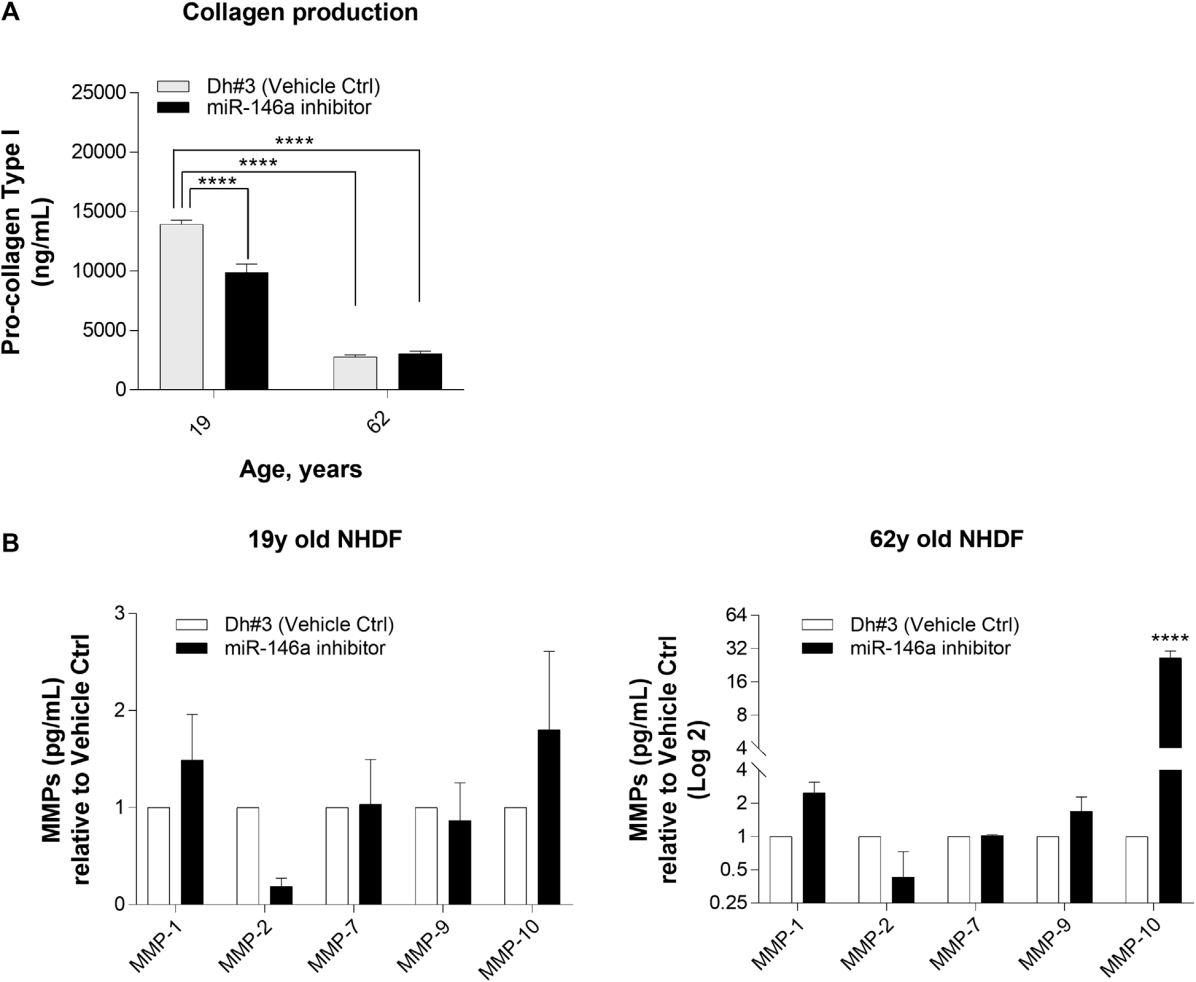
miR-146a inhibition impacts the release of pro-collagen type I and MMPs in NHDFs. **(A)** miR-146a inhibition leads to a decrease of pro-collagen type I in 19y old NHDF assayed by pro-collagen type I ELISA kit. **(B)** miR-146a inhibition in 62y old NHDF leads to a significant increase of MMP-1, MMP-9 and MMP-10 assayed by Multiplex ELISA measurement. ****p < 0.0001. Error bars ± SEM; Dh#3, Dharmafect #3; MMP, Matrix Metalloproteinase; Data are representative of at least two independent experiments.

We noted that inhibition of miR-146a alters collagen level and the expression of MMPs. Differences in collagen production, as measured by pro-collagen type I protein expression, were shown in 19y old NHDF, whereas inhibition of miR-146a did not impact further the loss of collagen level in 62y old NHDF (Figure 4A). In addition, miR-146a inhibition increases the expression of MMP-1, MMP-9, and MMP-10 in 62y old NHDF (Figure 4B).

#### DNA damage

**FIGURE 5:**
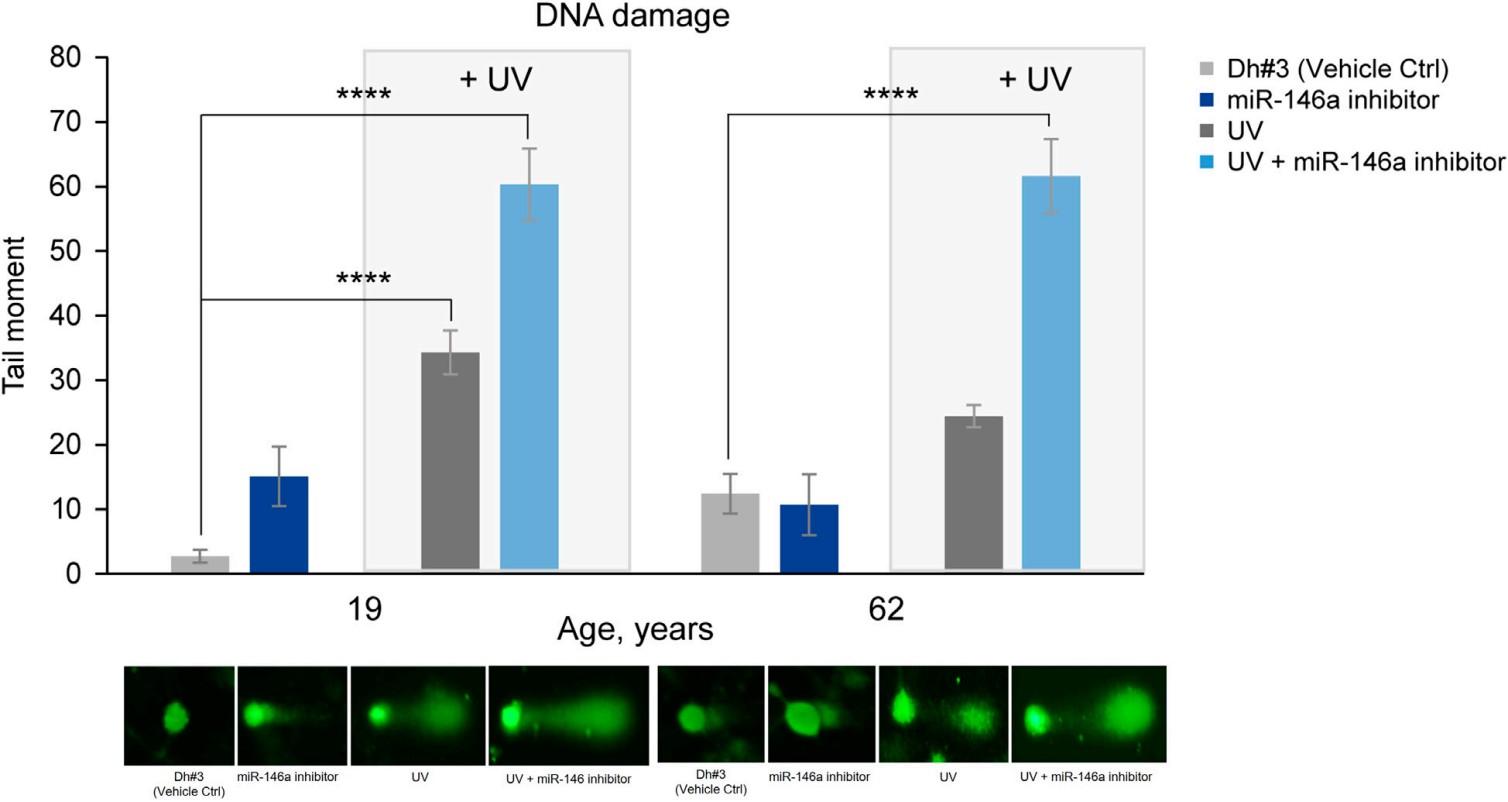
miR-146a inhibition increases DNA damage in NHDFs. miR-146a inhibition in 19y old and 62y old NHDFs leads to increased DNA damage (depicted in the graph as tail moment) measured by the comet assay and shown by the associated pictures beneath. ****p < 0.0001. Error bars ± SEM. Data are representative of at least two independent experiments.

DNA damage was assessed using the comet assay and reported as the tail moment. As expected, an increase in DNA damage was observed in 19y and 62y old NHDFs 6 hours post UV exposure. Pre-treatment of dermal fibroblasts with miR-146a inhibitor, before exposure to UV, significantly increased DNA damage in 19y and 62y old NHDFs. Interestingly, the miR-146a inhibition alone was sufficient to significantly increase the tail moment in 19y old NHDF, whereas no impact was observed in 62y old NHDF, potentially because miR-146a level is already significantly lower (Figure 5).

### Adansonia digitata extract can promote miR-146a expression and function

**FIGURE 6:**
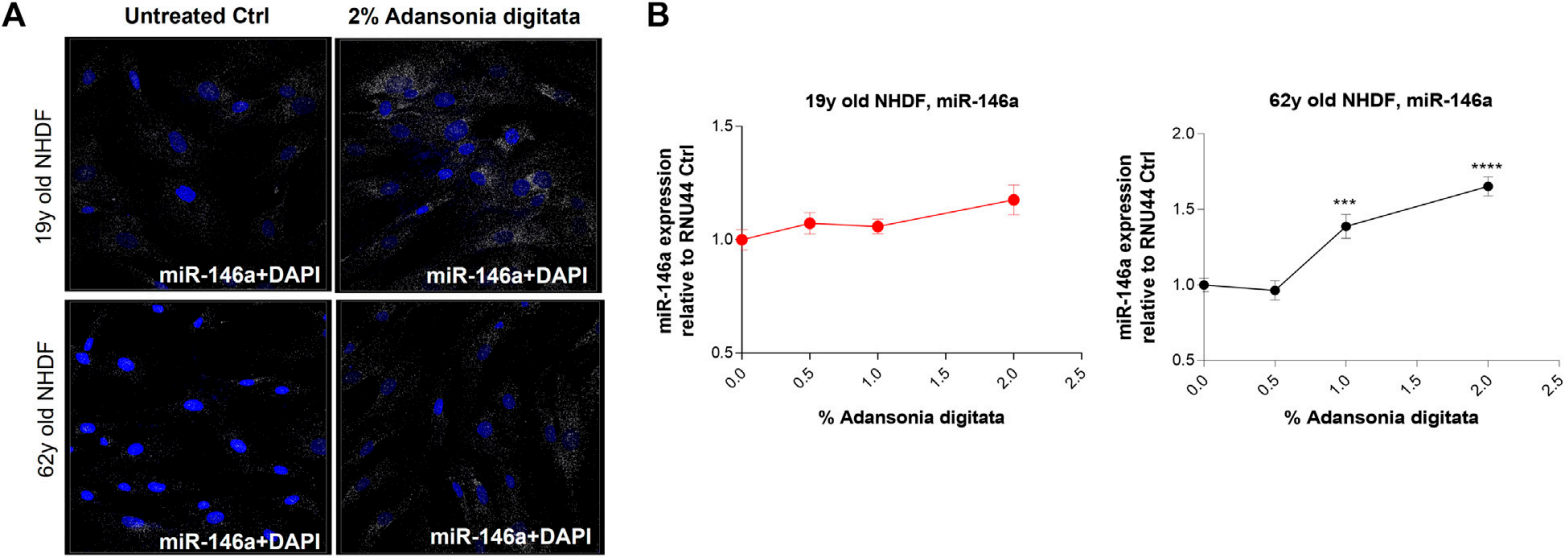
Adansonia digitata treatment increases the expression of miR-146a in NHDFs. **(A)** miR-146a expression was assayed by ViewRNA ISH assay before and after 2% Adansonia digitata treatment as shown by the associated confocal images. **(B)** miR-146a gene expression levels before and after treatments with Adansonia digitata were measured by qRT-PCR in 19y old and 62y old NHDFs. ***p = 0.001, ****p < 0.0001. ISH, *In situ* hybridization; Error bars ± SEM. Data are representative of at least three independent experiments.

Treatment with Adansonia digitata extract increased miR-146a levels in 62y old NHDF, partially restoring the expression seen in younger cells (Figure 6A). After 48 h exposure to different concentration of Adansonia digitata, the expression of miR-146a was evaluated by qRT-PCR. 62y old NHDF treated with 1% and 2% of Adansonia digitata showed a significantly greater increase of miR-146a expression than that of untreated cells (Figure 6B).

**FIGURE 7:**
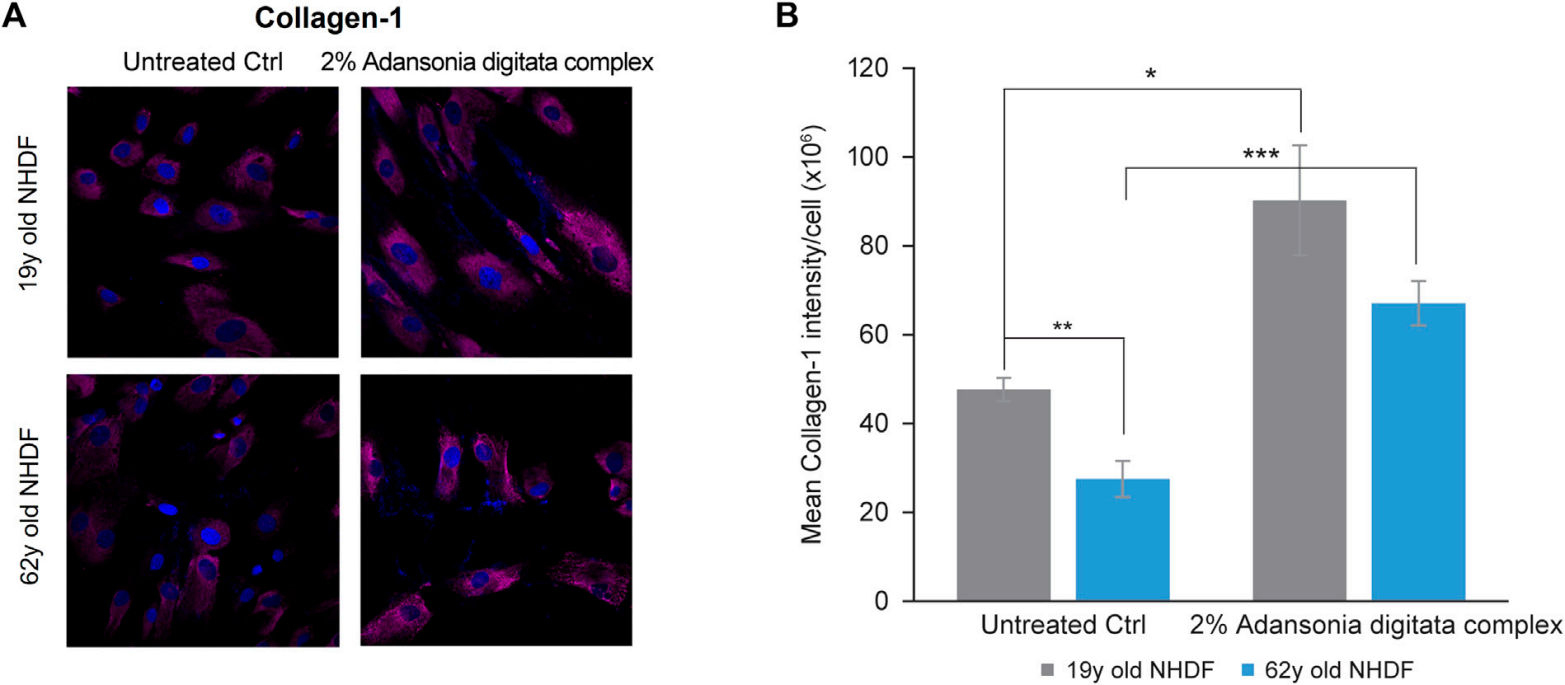
Adansonia digitata complex treatment increases the expression of Collagen-1 in NHDFs. **(A)** Collagen-1 expression in 19y old and 62y old NHDFs subjected to 2% Adansonia treatment complex for 72 h was assayed by ICC as shown by confocal microscopy pictures. **(B)** Mean intensity of Collagen-1 was calculated on both 19y old and 62y old NHDFs untreated and 2% Adansonia digitata complex-treated samples as depicted by the graph. **p = 0.01, ***p = 0.0001. ICC, Immunocytochemistry; Error bars ± SEM. Data are representative of at least two independent experiments.

Notably, several cellular processes that are deleteriously impacted by miR-146a inhibition could be addressed by treatment with Adansonia digitata extract. Specifically, Collagen-1 production was increased in 19y and 62y old NHDFs treated for 72 h with the Adansonia digitata complex, as assessed by immunofluorescence (Figure 7).

**FIGURE 8:**
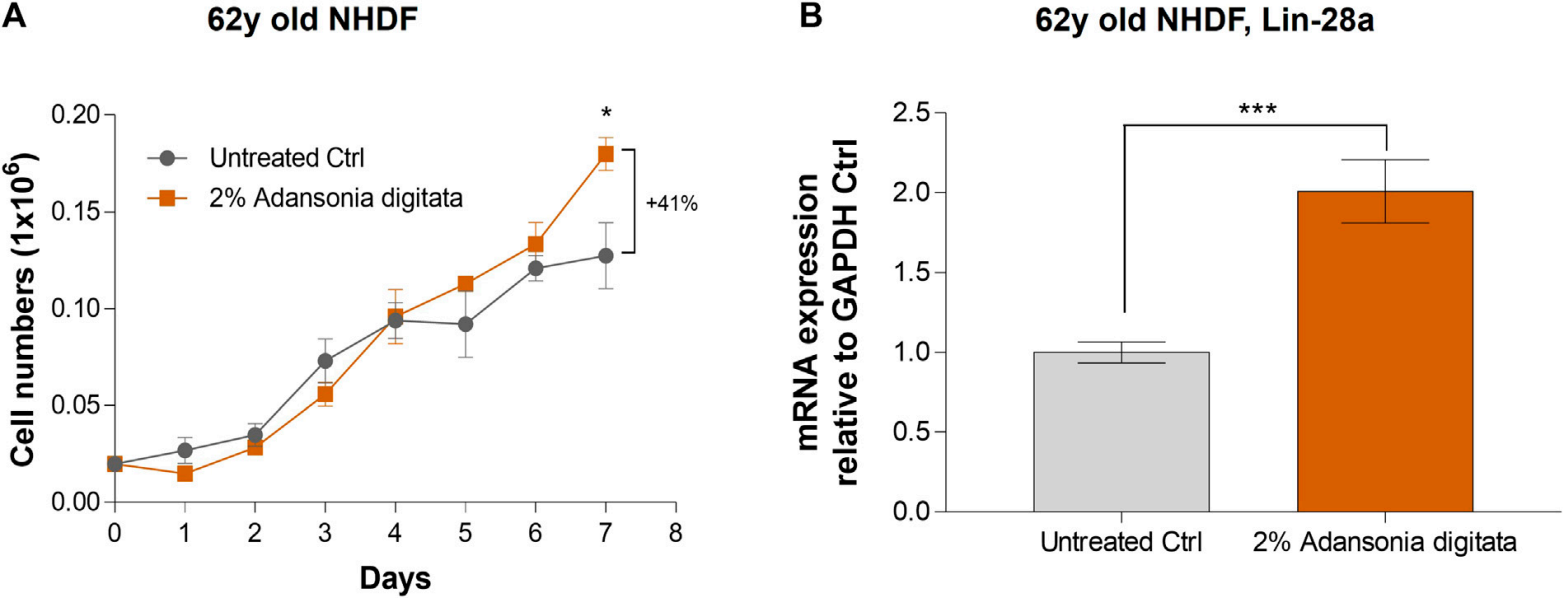
2% Adansonia digitata treatment increases cellular proliferation and Lin-28a gene expression levels in NHDFs. **(A)** Cellular proliferation of untreated 62y old NHDF and 2% Adansonia digitata-treated NHDF for 7 days was assayed by daily cell counts as depicted in the graph. **(B)** Lin-28a gene expression levels of untreated and 2% Adansonia digitata-treated samples were measured by qRT-PCR in 62y old NHDF. Error bars (±SEM). ***p = 0.001, *p < 0.05. Data are representative of at least two independent experiments.

**FIGURE 9:**
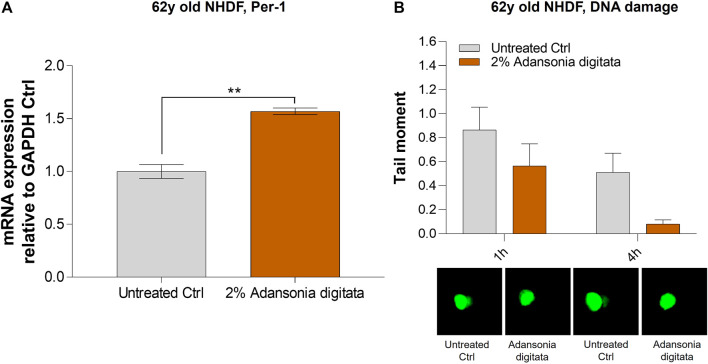
2% Adansonia digitata treatment increases the levels of Per-1 and lowers DNA damage in 62y old NHDF. **(A)** 62y old NHDF were subjected to 2% Adansonia digitata treatment and gene expression levels of Per-1 were assayed by qRT-PCR. **(B)** 62y old NHDF, treated with 2% Adansonia digitata and subjected to UV radiation exhibit reduced DNA damage (depicted in the graph as tail moment) measured by the comet assay as shown by the associated microscopy pictures beneath. **p = 0.01. Error bars ± SEM. Data are representative of at least two independent experiments.

In addition, cell proliferation was increased in 62y old NHDF after 7 days of treatment with Adansonia digitata extract (41% increase in cell number versus untreated cells, *p* = 0.033; Figure 8A). Furthermore, we observed a significant increase of the expression of Lin-28a and Per-1 in 62y old NHDF following Adansonia digitata extract treatment (Figure 8B; Figure 9A). Adansonia digitata treatment also significantly ameliorated DNA damage produced by UV exposure (Figure 9B).

### Adansonia digitata extract increases miR-146a in the exosome fraction

**FIGURE 10:**
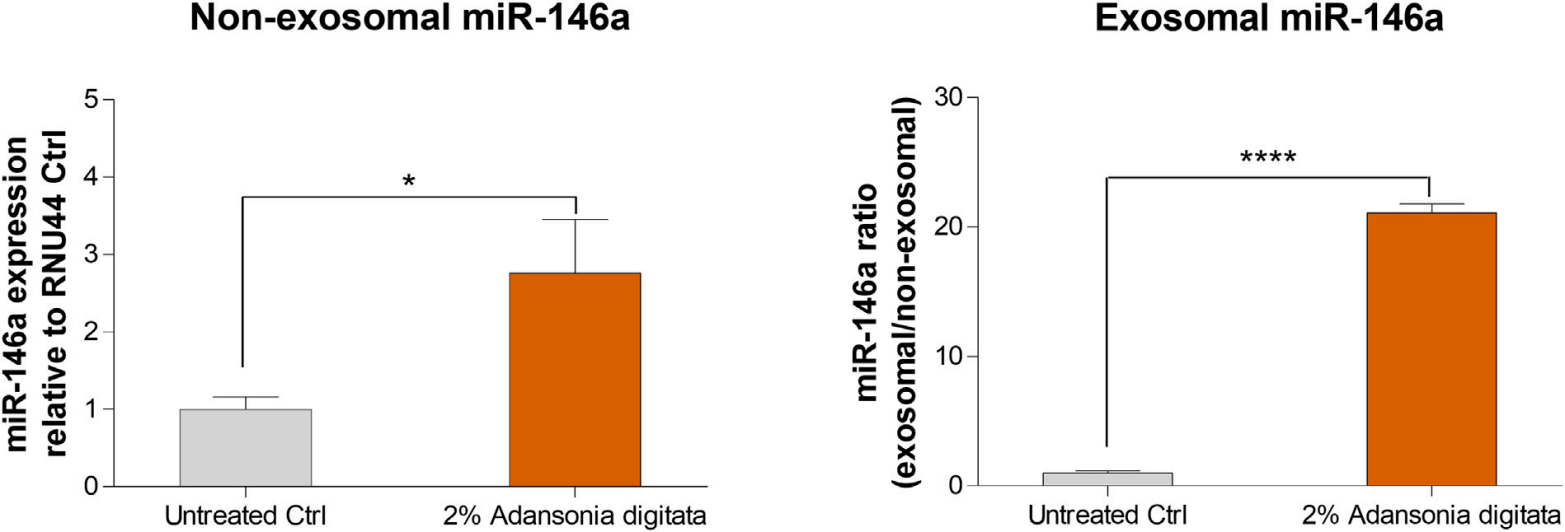
miR-146a is transported through exosomes in NHDFs. Naïve 62y old NHDF were incubated with the media of 19y old NHDF previously treated with 2% Adansonia digitata. Gene expression levels of non-exosomal (cellular fraction) and exosomal (supernatant fraction) miR-146a were assayed by qRT-PCR. *p < 0.05, ****p < 0.0001. Error bars ± SEM. Data are representative of at least three independent experiments.

Finally, we evaluated if miR-146a was transported through exosomes as previously shown (Hijmans et al., 2018; Orioli and Dellambra, 2018). 19y old NHDF were treated with 2% Adansonia digitata in exosome depleted FBS media for 48 h. Then, conditioned supernatants were transferred to naïve 62y old NHDF and after 24 h, the expression of miR-146a was evaluated in the non-exosomal (cellular fraction) and exosomal fraction (supernatant fraction). A significant increase of miR-146a was observed in the non-exosomal fraction and a dramatic increase was observed in the exosomal fraction (Figure 10). Altogether these data demonstrate that miR-146a is transported between cells via exosomes and that the treatment with Adansonia digitata drastically increases miR-146a expression in the exosomal fraction.

## Discussion

Skin is the largest organ of the human body, accounting for ∼15% of total body weight (Livak and Schmittgen, 2001; Kanitakis, 2002), and its physiological functions are regulated by epigenetic mechanisms including miRNAs among others. The seminal discovery of miRNAs in the nematode *C. elegans* in 1993 by Lee and colleagues opened the door to a completely new field of developmental biology (O’Connell et al., 2012), redefining longstanding dogmas of gene regulation (Healy, 2005). Within skin, a diverse pool of miRNAs orchestrates physiological functions central to the maintenance of tissue homeostasis and health. This regulatory machinery can be perturbed by exposure to exogenous stressors such as UV irradiation. It has also been well documented that chronological aging is associated with an accumulation of damage, in particular an increase in hallmarks of inflammation-associated aging or “inflammaging” that slow down recovery and renewal (Franceschi et al., 2018).

After a literature review and initial experimental screening of various miRNAs involved in skin aging, circadian rhythm control and regulation of crucial pathways that promote skin health, miR-146a-5p (miR-146a) was identified. To validate the finding of the initial screening we focused on NHDF cells and found that miR-146a expression decreased with age (Figures 1A, B; Supplementary Figure S1) in both Caucasian and Chinese donors respectively. *In vivo* data from clinical studies focused on patients affected by comorbidities such obesity demonstrated a correlation with lower levels of miR-146a and miR-150 circulating in the plasma. This suggests that dysregulation of these miRNAs could be associated with the heightened inflammatory state of these pathologies (Hijmans et al., 2019). Other studies reported that lower levels of miR-146a were found in patients with sleep abnormalities (e.g., short sleepers vs. normal sleepers) (Wang et al., 2014), suggesting that chronic short sleep was associated with reduced miR-146a levels with its relation to circadian rhythm machinery that could in turn exacerbate inflammation. In addition, studies in both diabetic mice and human retinal endothelial cells showed that diabetic retinopathy leads to alterations in oscillations of miR-146a levels, thereby impacting the expression of both the circadian clock genes Per1 and Bmal-1 (Srivastava et al., 2017). Here, we investigate whether miR-146a plays a role in the regulation of circadian rhythm and clock genes in human skin. To begin, we inhibited miR-146a in young and aged fibroblasts and found that there was a statistically significant decline of Bmal-1 in young (19y old NHDF) cells, and a decline of Bmal-1, Per-1 and Clock in aged (62y old NHDF) cells respectively (Figure 2). The importance of miR-146a to many other cellular processes has been reported including inflammation (O’Connell et al., 2012; Salido-Guadarrama et al., 2014; Boldin et al., 2011; Rebane et al., 2014), senescence (Bhaumik et al., 2009), hematopoiesis (Zhao et al., 2013), and cellular proliferation (Qiu et al., 2020) etc.; however, a clear connection with skin has yet to be made. Here, we show that inhibition of miR-146a could impact cellular proliferation and inflammation. In tracking cell number for young and aged cells over the course of 5 days (Figure 3A; Supplementary Figure S3) we also show evidence of impairment of cell proliferation following miR-146a inhibition. There is a preponderance of evidence in literature that miR-146a contributes to the regulation of NF-kB (Zhou et al., 2019). In tracking a panel of inflammatory cytokines following miR-146a inhibition (Figure 3B), we found that 62y old NHDF cells showed a significant elevation of multiple pro-inflammatory mediators, including IL-6, IL-8, IL-12P70, IL-17α, TNF-α. This is consistent with an anti-inflammatory role for miR-146a within the skin (O’Connell et al., 2012).

When skin is exposed to environmental insults (e.g., UV irradiation or oxidative damage), there is a consequent increase in the expression of matrix metalloproteinases (MMPs) (Pittayapruek et al., 2016). MMPs are ubiquitous endopeptidases that degrade the extracellular matrix components such as Collagen, Elastin, and Fibronectin (Van Doren, 2015; Shin et al., 2019). We determined that miR-146a inhibition resulted in a slight increase in MMP-1 (Collagenase) and MMP-10 (Stromelysin) in young NHDF cells, and a more robust increase in MMP-1 (Collagenase), MMP-9 (Gelatinase) and MMP-10 (Stromelysin) in the older NHDF cells (Figure 4). Inhibition of miR-146a led to an increase of MMP-1 levels and to a statistically significant loss of Collagen-1 in 19y old NHDF cells. The loss of Collagen-1 was much more pronounced in the 19y old NHDF cells as compared to 62y old NHDF cells, possibly due to the loss of collagen production with age (Figure 4).

When skin is exposed to UVA and UVB irradiation, DNA damage and an increase in oxidative damage occur (Kciuk et al., 2020). Inhibiting miR-146a in both 19y old and 62y old NHDF cells prior to UV exposure led to statistically significant increase in DNA damage as shown by the tail moment in a comet assay (Figure 5).

In an effort to ameliorate the multiple deleterious physiological effects seen in skin cells as a result of miR-146a downregulation, we treated cells with an extract derived from the Baobab tree (Adansonia digitata), commonly found in the thorn woodlands of African savannahs (Rahul et al., 2015). Baobab has been shown to have a plethora of biological properties such as antiviral, antioxidant and anti-inflammatory, and various parts of the plant have been traditionally used to fight diseases and treat numerous ailments (Rahul et al., 2015). For example, baobab fruit has been touted as a “super fruit” for its nutritional profile which includes a high content of Vitamin C in the pulp (280–300 mg/100g), as well as high levels of minerals such as Calcium, Magnesium and Phosphorus (Rahul et al., 2015). We assessed qualitatively the basal levels of miR-146a in cells before and after the treatment (Figure 6A) and found that we could significantly elevate miR-146a expression in aged skin cells (Figure 6B). We then treated the cells with complex containing Adansonia digitata and assessed induction of Collagen-1 expression by ICC (Figure 7A). Quantification of the Collagen-1 imaging data showed a statistically significant increase following treatment (Figure 7B).

Our findings demonstrated that miR-146a inhibition impacted cellular proliferation in fibroblasts and keratinocytes (Figure 3; Supplementary Figure S3). Since the most robust effects were seen in aged cells, we cultured the 62y old NHDF with Adansonia digitata and measured proliferation for 1 week, finding an increase at day 7 as shown in Figure 8A. To see if other proliferation targets could be impacted by Adansonia digitata treatment we measured the expression of Lin-28a (Figure 8B), an RNA-binding protein that regulates stem cell differentiation and proliferation (Wu et al., 2022). Recent studies have also linked Lin-28a to wound healing (Ng et al., 2013), tissue repair (Yuko et al., 2021) and metabolic reprogramming of mitochondria (Pieknell et al., 2021). Since miR-146a inhibition impacted the clock gene machinery, we treated our cells with Adansonia digitata extract, and demonstrated a consequent increase of Per-1 levels (Figure 9A). Adansonia treatment additionally lowered DNA damage 4 hours post-exposure to UV irradiation, demonstrated by a reduction in the tail moment of the comet assay (Figure 9B).

Previous literature findings showed that miR-146a could be transported through exosomes (Alexander et al., 2015; Fu et al., 2017). Exosomes are extracellular vesicles (EVs) that shuttle molecular cargoes from donor cells to recipient cells and play a crucial role in mediating intercellular communication (Yu et al., 2022). We treated young NHDF cells with our extract, isolated the non-exosomal and exosomal miR-146a fractions and we found that Adansonia digitata treatment led to a statistically significant transfer of miR-146a in aged cells (Figure 10). Besides the transfer of miR-146a through exosomes, the media could contain chemokines, growth factors and other mRNAs that could contribute to the increased expression of miR-146a in the aged cells. Exosomes hold great promise to serve as novel biomarkers in liquid biopsy to fight cancer for precision medicine, and speculatively this raises the possibility that the exosomes taken from younger or conditioned cells could be leveraged to induce positive biological effects in older cells therefore recapturing a more youthful phenotype.

Taken together, we showed that the loss of miR-146a in skin cells contribute to the aging process where it results in a loss of synchronization with circadian rhythm, repair efficiency as well as protein production and cellular proliferation with an increase of damaging factors such as inflammatory mediators and MMPs. With an Adansonia digitata extract, we are able to re-establish a miR-146a level closer to young skin cells and help mature skin cells to recover key functions to repair and rebuild a strong skin. As well, due to its exosomal transport, miR-146a will be able to move from one cell to another to amplify this positive benefit.

Overall, these results have shown the importance of miR-146a to fight key hallmarks of aging in the skin.

## Conclusion

These findings support that miR-146a is a key mediator of biological pathways related to fighting aging in skin cells; particularly associated with circadian rhythm, inflammation, cell-to-cell communication, cell damage repair, and proliferation.

## Data Availability

The raw data supporting the conclusion of this article will be made available by the authors, without undue reservation.

## Supplementary material

SUPPLEMENTARY FIGURE S1 Expression of miR-146a in Chinese NHDFs decreases with age. miR-146a expression of 21y, 27y, and 40y old NHDFs relative to RNU44 Ctrl assayed by qRT-PCR. **P* < 0.05, Error bars ± SEM; Data are representative of at least three independent experiments.

SUPPLEMENTARY FIGURE S2 miR-146a inhibition reduces the expression of miR-146a in NHDFs. miR-146a gene expression levels were measured by qRT-PCR in 19y old and 62y old NHDFs. Error bars ± SEM; Data are representative of at least two independent experiments.

SUPPLEMENTARY FIGURE S3 miR-146a inhibition impacts cellular proliferation of NHEK. Cell count of adult NHEK subjected to five days of miR-146a inhibition. Cell count was performed each day post treatment on both Dh#3 (Vehicle Ctrl) and miR-146a inhibitor-treated samples. ****P* = 0.001, Dh#3, Dharmafect #3; NHEK, normal human epidermal keratinocytes; Error bars ± SEM. Data are representative of at least two independent experiments.
