# Supplementary figures and images for: miR-146a is a critical target associated with multiple biological pathways of skin aging
Source: Front Physiol. 2024 Feb 29;15:1291344. doi: 10.3389/fphys.2024.1291344 (PMC10937357; doi:10.3389/fphys.2024.1291344)

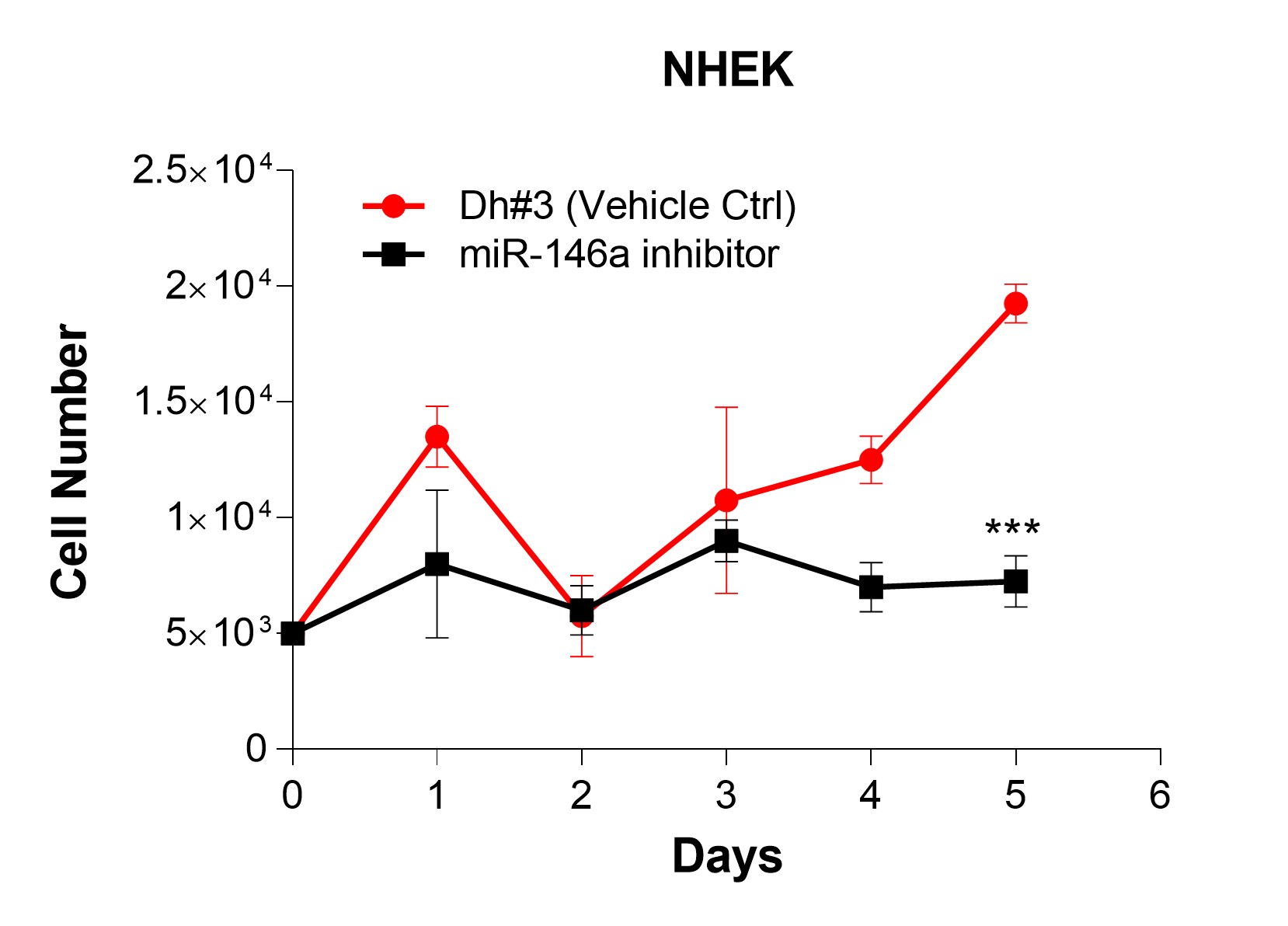

Supplement: Supplementary file 1 [file Image3.jpg]

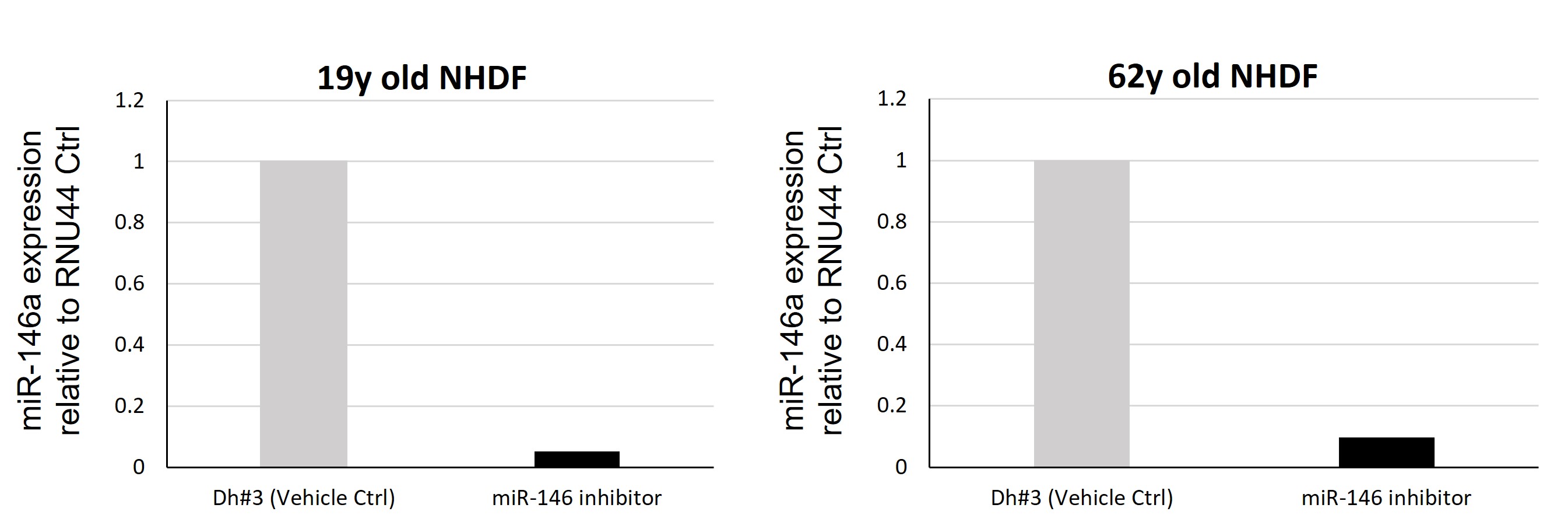

Supplement: Supplementary file 2 [file Image2.jpg]

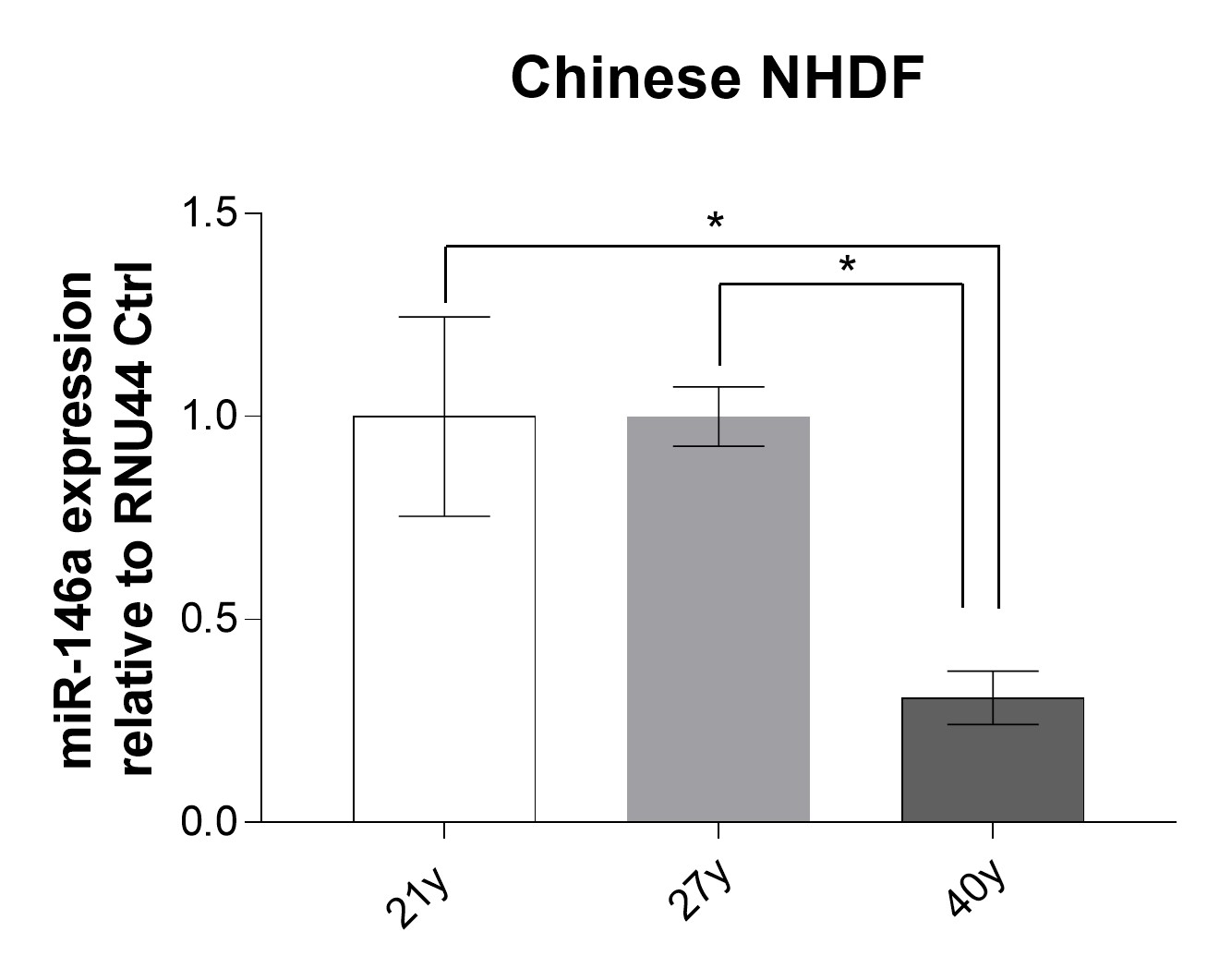

Supplement: Supplementary file 3 [file Image1.jpg]
